# Pleural IFN-γ release assay combined with biomarkers distinguished effectively tuberculosis from malignant pleural effusion

**DOI:** 10.1186/s12879-018-3654-z

**Published:** 2019-01-16

**Authors:** Yimin Tang, Juanjuan Zhang, Huarong Huang, Xing He, Jiaohong Zhang, Min Ou, Guobao Li, Changchun Zeng, Taosheng Ye, Lili Ren, Yingxia Liu, Guoliang Zhang

**Affiliations:** 1 Department of Tuberculosis, Shenzhen Third People’s Hospital, University of South China, Shenzhen, 518112 China; 2 Department of Laboratory Medicine, Shenzhen Longhua District Central Hospital, Guangdong Medical University, Shenzhen, 518110 China; 3 Guangdong Key Laboratory of Emerging Infectious Diseases, Shenzhen Third People’s Hospital, University of South China, Shenzhen, 518112 China; 4 MOH Key Laboratory of Systems Biology of Pathogens and Christophe Mérieux Laboratory, IPB, CAMS-Fondation Mérieux, Institute of Pathogen Biology (IPB), Chinese Academy of Medical Sciences & Peking Union Medical College, Beijing, 100730 China

**Keywords:** Tuberculosis pleural effusion, Malignant pleural effusion, IFN-γ release assay, Adenosine deaminase, Carcinoembryonic antigen

## Abstract

**Background:**

Tuberculosis (TB) remains a major public health concern on a global scale, especially in developing nations. So far, no formal guidelines are available for the diagnosis and treatment of tuberculosis pleurisy. The diagnosis of TB is worsened by the immense difficulty in differential determination of tuberculosis pleural effusion (TPE) and malignant pleural effusion (MPE). The purpose of this investigation is to assess the differential diagnostic efficiencies of the pleural IFN-γ release assay (IGRA) and widely-used biochemical parameters in the distinction analysis of TPE and MPE.

**Methods:**

A cohort of 222 patients with pleural effusion was examined, comprising of 143 TPE and 58 MPE patients. The patients were examined with IGRA, and the widely-used biomarkers in the pleural effusion and peripheral blood.

**Results:**

Our results show that the TPE patients have significantly higher *M. tuberculosis* (Mtb) antigen-specific IFN-γ responses to ESAT-6 protein and peptide pool in the blood compared to MPE patients. TPE patients were also shown to have enriched Mtb antigen-specific IFN-γ responses in pleural effusion than in peripheral blood. Among the widely-used biomarkers, the adenosine deaminase (ADA) and carcinoembryonic antigen (CEA) in pleural effusion were better biomarkers with high sensitivity and specificity to discriminate TPE and MPE. In addition, pleural IGRA could not be affected by the pleural adhesion, and the applications of the pleural IGRA together with ADA and CEA provide a promising approach for the TPE and MPE differential identification.

**Conclusions:**

Our study proposes that the integration of pleural IGRA and ADA, CEA detection could add to more effective diagnosis stratagems in the discernment between TPE and MPE.

## Background

Tuberculosis (TB) accounts for a major public health issue concerning millions of people in both developed and developing countries. Tuberculosis pleural effusion (TPE) represents the most common factor of pleural effusion in regions where TB is endemic, and the second most predominant type of extrapulmonary tuberculosis (after lymphatic involvement) [1]. TPE usually manifests as an acute disease with cough, fever and pleuritic chest pain. It results from pleural infection by *M. tuberculosis* (Mtb) and is characterized by intense build-up of fluid and inflammatory cells in pleural cavity [2]. The definitive TPE diagnosis is the Mtb detection in pleural fluid, sputum, and/or pleural biopsy specimens [3]. However, the effusions can cause postponed hypersensitivity reaction to antigens of mycobacteria, leading to prolonged delay and/or false negative results in microbiological analyses [4].

In the past decades, many bio-parameters have provided simple and cost-effective methods for tuberculosis pleurisy diagnosis. Among all, adenosine deaminase (ADA) has been described to be a presumptive test for the early diagnosis of TPE, where the specimens have to demonstrate ADA level above the cutoff value of 40 U/L [5]. ADA is a major T-lymphocyte enzyme that catalyzes the conversion of adenosine and deoxyadenosine to inosine and deoxyinosine [6]. Many studies have described ADA level as sensitive and specific biomarker for diagnosing TPE [7, 8]. However, high pleural effusions ADA level are also associated with the other etiologies, such as empyemas and parapneumonia [9]. In addition, high pleural fluid ADA has been documented in brucellosis, Q fever, lymphomas, and rheumatoid arthritis [10]. In the early phase of the TPE, low levels of ADA may be found, resulting in false negative diagnosis. Therefore, there is likelihood of both the false-negative and false-positive results using the ADA diagnostic method.

On the other hand, cancer contributes to a major factor of pleural effusions, namely malignant pleural effusion (MPE) [11]. Affecting mostly lung or breast cancer, MPE is characterized by the manifestation of neoplastic cells in the pleural region. MPE is frequently misdiagnosed as TPE due to the similar clinical characteristics, including lymphocytic pleural exudates, which represent the main challenge in distinguishing the pleural effusions from each other [12]. Patients with confirmed MPE have completely different prognosis and clinical treatment, to that of TPE. Thus, an accurate diagnosis should be performed on patients with symptomatic pleural effusions, to allow the initiation of efficient management strategy for pleural TB. The current study was carried out to perform the comparison of biomarkers in peripheral blood and pleural effusion to discriminate TPE and MPE, furthermore, the combinations of pleural IGRA with ADA, carcinoembryonic antigen (CEA) were investigated for a potentially differential diagnostic algorithm.

## Methods

### Study population

A total of hospitalized 222 patients were enrolled in this investigation at Shenzhen Third People’s Hospital from January 2010 to December 2016. Among these patients, 21 patients were diagnosed with pneumonia or other diseases, and were excluded from further studies. Based on the examination, 201 patients with pleural effusion were categorised into 2 categories: the TPE category (*n* = 143) and the MPE category (*n* = 58), as tabulated in Table 1. All of the participants were HIV negative.

**Table 1:** The clinical characteristics and demographic of study participants

	Total	TPE	MPE
Patients, (n)	201	143	58
Median age (range)	37 (6–85)	31 (6–83)	52 (28–85)
Male (%)	131 (65.17)	99 (69.23)	32 (55.17)
Positive, sputum, AFB smear or culture, n (%)	_	56/143 (39.16)	0
Mtb detection in pleural effusion	_		
Positive AFB smear, n (%)	_	1/143 (0.70)	0
Positive culture, n (%)	_	17/143 (11.89)	0
Confirmed TPE by pleural biopsy, n (%)		60/78 (76.92)	0

### Diagnostic measures for the pleural effusion

The pleural effusions were initially detected as exudates with Light’s criteria. Established TPE was identified by the manifestation of positive Mtb culture in pleural effusion using the Bactec MGIT 960 culture system per the manufacturer’s instructions, and/or pleural biopsy specimens positive for granulomatous inflammation with acid-fast bacilli (AFB) present. Probable TPE was diagnosed by either of the criteria as follows: positive Mtb culture in sputum, or positive reaction to anti-tuberculosis drug without other potential sources of pleural effusion. MPE was considered when positive pleural fluid cytology and/or biopsy histology was observed.

### IFN-γ release assay in peripheral blood and pleural effusion

Mtb-specific IFN-γ production by peripheral blood mononuclear cells (PBMCs) and pleural fluid mononuclear cells (PFMCs) was determined by using an in-house ELISPOT assay as described previously [13, 14]. In brief, 2 × 10^5^ fresh PBMCs or PFMCs were plated overnight on 96-well plates precoated with a mouse anti-human IFN-γ antibody, which were left unstimulated (negative control) or were stimulated with phytohemagglutinin (PHA, positive control) and the different Mtb-specific antigens at a final concentration of 10 μg/ml (ESAT-6 protein and ESAT-6/CFP-10 peptide pool) for 24 h. Recombinant ESAT-6 was expressed in *Escherichia coli* and purified with Ni + affinity chromatography. ESAT-6/CFP-10 peptides with 20 amino acids in length were synthesized by Hanyu Company (Shenzhen, China). After washing of the plates, a conjugate incubation followed by a detection step was carried out to visualize the spot forming cells (SFCs) using an automated image analysis system ELISPOT reader (BioReader 4000 Pro-X, Biosys, Germany). The number of SFCs in response to ESAT-6 protein and peptide were determined after subtracting the number of SFCs in the negative control well, and the results were considered positive if the net numbers were over the cutoff value as determined by receiver operating characteristic (ROC) analysis.

### Determination of the ADA, CEA, TP, and LDH levels in pleural effusion

ADA levels were measured in pleural effusion by peroxidase method using a commercial kit (Purebio, Ningbo, China). CEA levels were determined by direct chemilumiscence immunoassay using a kit from Siemens (Germany). The levels of total protein (TP) and lactic dehydrogenase (LDH) were detected in the clinical laboratory of Shenzhen Third People’s Hospital using a HITACHI 7020 Automatic Biochemical Analyzer, and the kits were manufactured by Kefang Biological Technology (Guangzhou, China).

### Statistical analysis

The difference between two groups of TPE and MPE was analyzed by unpaired t test, the matched Mtb antigen-specific IFN-γ responses in PBMCs and PFMCs were analyzed by paired t test. ROC analysis was performed to determine the diagnostic accuracies of biomarkers and Mtb antigen-specific IFN-γ responses to distinguish patients with TPE from those with MPE. The cutoff values were identified at multiple specificities and sensitivities, and assessed at the maximum Youden’s index (YI). Differences were considered significant when a two-tailed *P* value was less than 0.05. The data was analyzed using GraphPad Prism V5.0 software (San Diego, CA, USA).

## Results

### Clinical features of study participants

The recruited 201 patients have a median age of 38 years. The patients with MPE were elder than the TPE patients (median 54.5 vs 31 years, *P* < 0.01). Of the patients enrolled, 65.2% made up of male (131 out of the 201 patients). Among the 143 patients with TPE, 78 patients were identified with established tuberculosis pleurisy with culture and biopsy evidences: 1 by positive AFB smear, 17 by positive pleural culture, and 60 by pleural biopsy. The other 65 patients with TPE were identified with apparent tuberculosis pleurisy with clinical evidence as a result of positive culture of Mtb in the sputum or positive responses to anti-TB treatment. All of the MPE 58 patients were histologically identified by bronchoscopy or thoracoscopy.

### Diagnostic performance of IGRA

**Fig. 1:**
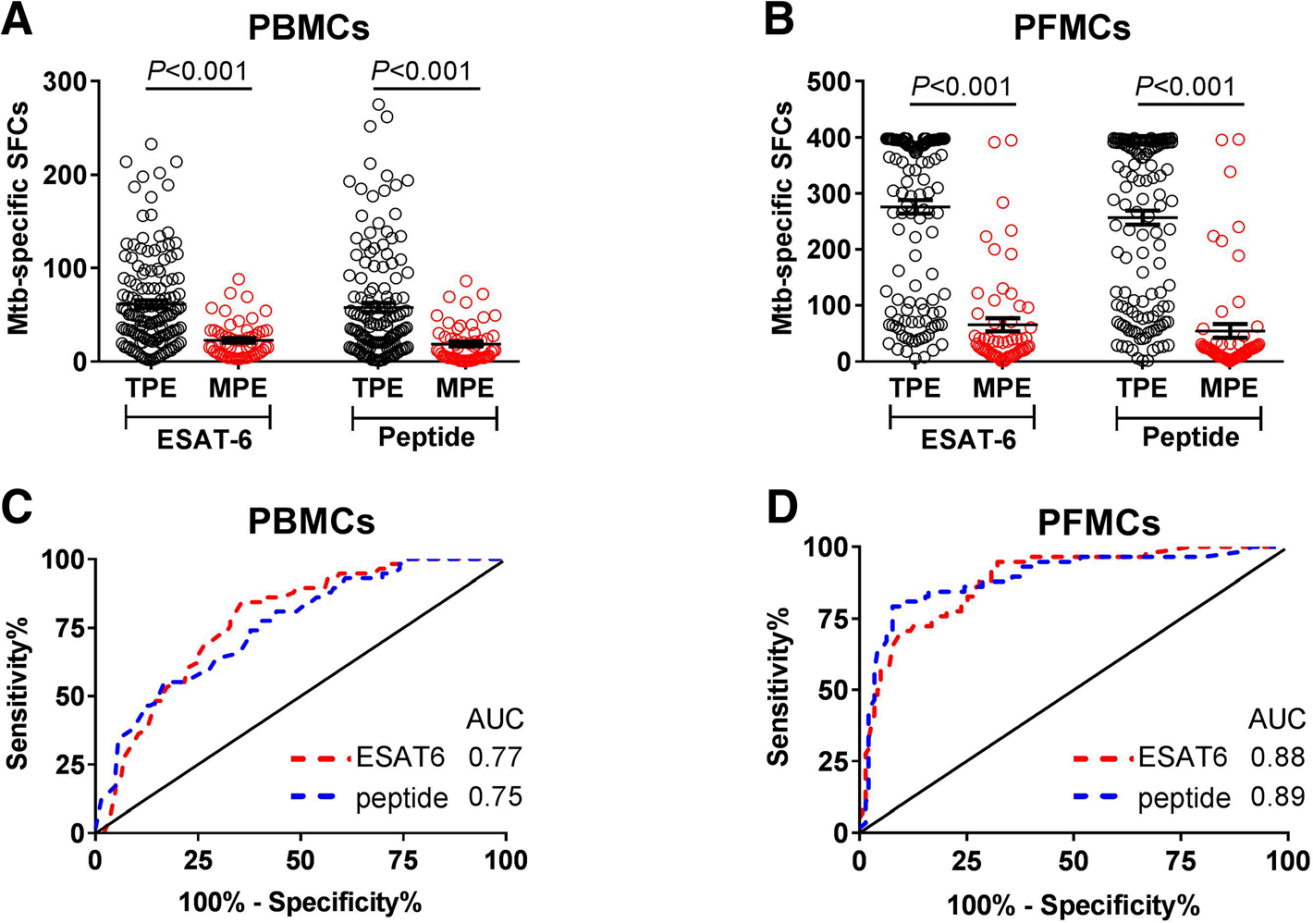
The PFMCs IGRA shows higher diagnostic performance to discriminate TPE and MPE. **a** The PBMCs from TPE (*n* = 143) and MPE (*n* = 58) patients were stimulated with ESAT-6 protein and ESAT-6/CFP-10 peptide pool, after 24hs incubation, IFN-γ-producing cells were determined using ELISPOT reader. **b** The PFMCs isolated from pleural effusion were stimulated with ESAT-6 and peptide pool, and the differences of SFCs were compared between TPE (*n* = 143) and MPE (*n* = 58) patients. The unpaired t test was used for the analysis. **c**, **d** ROC analysis was conducted to determine the power of IGRA in discriminating TPE and MPE in PBMCs (**c**) and PFMCs (**d**). The AUC of both ESAT-6 and peptide induced IFN-γ responses was determined

The responses of Mtb antigen-specific IFN-γ in peripheral blood and pleural effusion were performed for all the patients. As shown in Fig. 1, Mtb antigen-specific (ESAT-6 and ESAT-6/CFP-10 peptide pool) IFN-γ-producing cells in PBMCs were significantly higher in TPE than that in MPE (Fig. 1a), and similar pattern was also observed in PFMCs (Fig. 1b). The ROC curve study presented the area under the curve (AUC) of the antigen specific ESAT-6 and peptide for distinguishing TPE and MPE to be 0.77 and 0.75, respectively in PBMCs (Fig. 1c), whilst they were found to be 0.88 and 0.89 in PFMCs (Fig. 1d) respectively, suggesting that performance of the PFMCs IGRA is significantly improved compared with that of PBMCs IGRA.

### Elevated Mtb specific IFN-γ responses in PFMCs

**Fig. 2:**
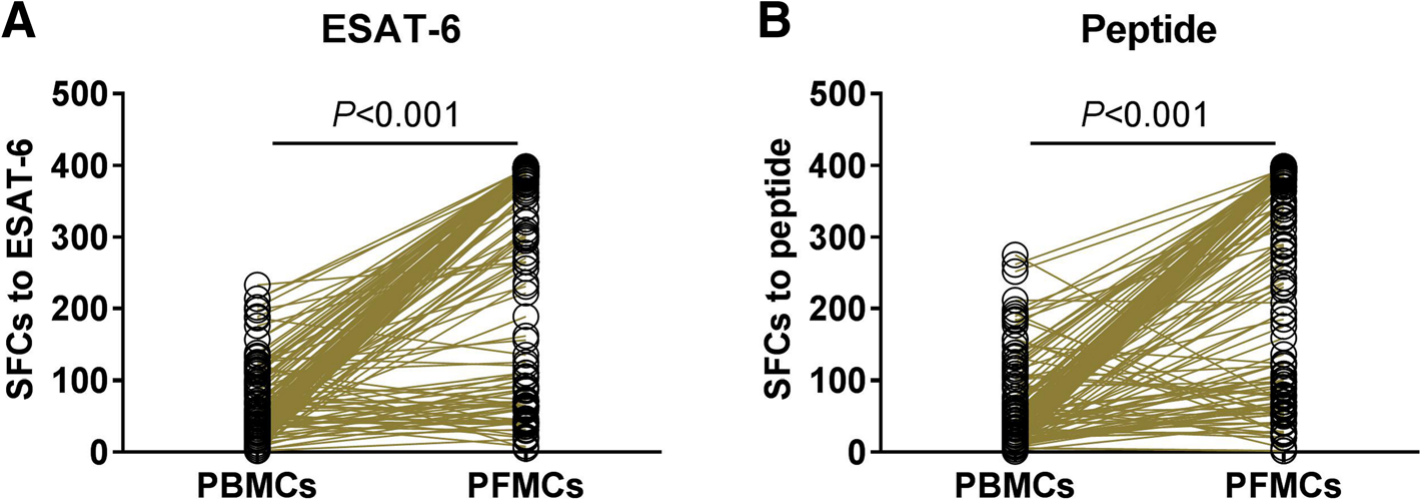
Mtb elicits elevated IFN-γ responses in PFMCs than PBMCs in TPE patients. The matched PBMCs and PFMCs from TPE (*n* = 143) patients were stimulated with ESAT-6 protein (**a**) and peptide (**b**), and Mtb-specific IFN-γ responses were determined using ELISPOT reader. The paired t test was used to compare the difference

To further compare the difference of Mtb specific IFN-γ responses in peripheral blood and pleural effusion, the matched PBMCs and PFMCs were used to determine the numbers of IFN-γ producing cells after ESAT-6 and peptide stimulation. As expected, it was observable that antigen specific IFN-γ responses was significantly elevated (*P* < 0.001) when tested in PFMCs, to that of PBMCs, no matter with ESAT-6 (Fig. 2a) or peptide (Fig. 2b) stimulation. These results suggest that Mtb infection elicits stronger IFN-γ responses at the site of infection.

### Assessment of biomarkers in pleural effusion

In assessment of analytical potential for TPE and MPE identification, we assessed the enrolment variables of the participants from blood tests and effusion tests in the two categories. As presented in Table 2, LDH, monocyte %, TP and GLU in the effusion were not different between both categories, whilst erythrocyte sedimentation rate (ESR), C-reactive protein (CRP) in peripheral blood and white cell count (WCC) in the effusion exhibited significant difference between TPE and MPE category. However, ROC curve analysis showed that the AUC of these 3 potential biomarkers was lower than IGRA in PBMCs and PFMCs.

**Table 2:** The comparisons of biomarkers in peripheral blood and pleural effusion between TPE and MPE

	TPE median (90%range)	n	MPE median (90% range)	n	*P* value	AUC (95%CI)
Blood tests						
ESR (mm/h)	57 (26.30–97.70)	136	35 (5.30–72.90)	52	< 0.001	0.73 (0.65–0.81)
CRP (mg/L)	79.54 (25.10–130.70)	126	17.15 (2.90–84.41)	30	< 0.001	0.75 (0.67–0.84)
Effusion tests						
WCC (10^6^/L)	3989 (580.80–10,544)	91	1772 (504.60–3966)	58	< 0.001	0.74 (0.64–0.84)
Monocytes (%)	76.25 (39.90–91.10)	139	76.55 (38.20–91.20)	58	0.30	0.55 (0.46–0.63)
LDH (IU/L)	689.52 (336.30–1226.15)	128	496 (179.11–2007.32)	58	0.15	0.57 (0.47–0.67)
TP (g/L)	53.56 (44.90–60.51)	91	49.44 (37.00–65.50)	58	0.02	0.61 (0.51–0.71)
GLU (mmol/L)	4.25 (2.00–6.00)	91	4.86 (0.20–7.70)	58	0.09	0.58 (0.48–0.68)

Abbreviations: *ESR* erythrocyte sedimentation rate, *CRP* C-Reactive protein, *WCC* white cell count, *LDH* lactate dehydrogenase, *TP* total protein, *GLU* glucose

### Comparison of pleural ADA and CEA level

**Fig. 3:**
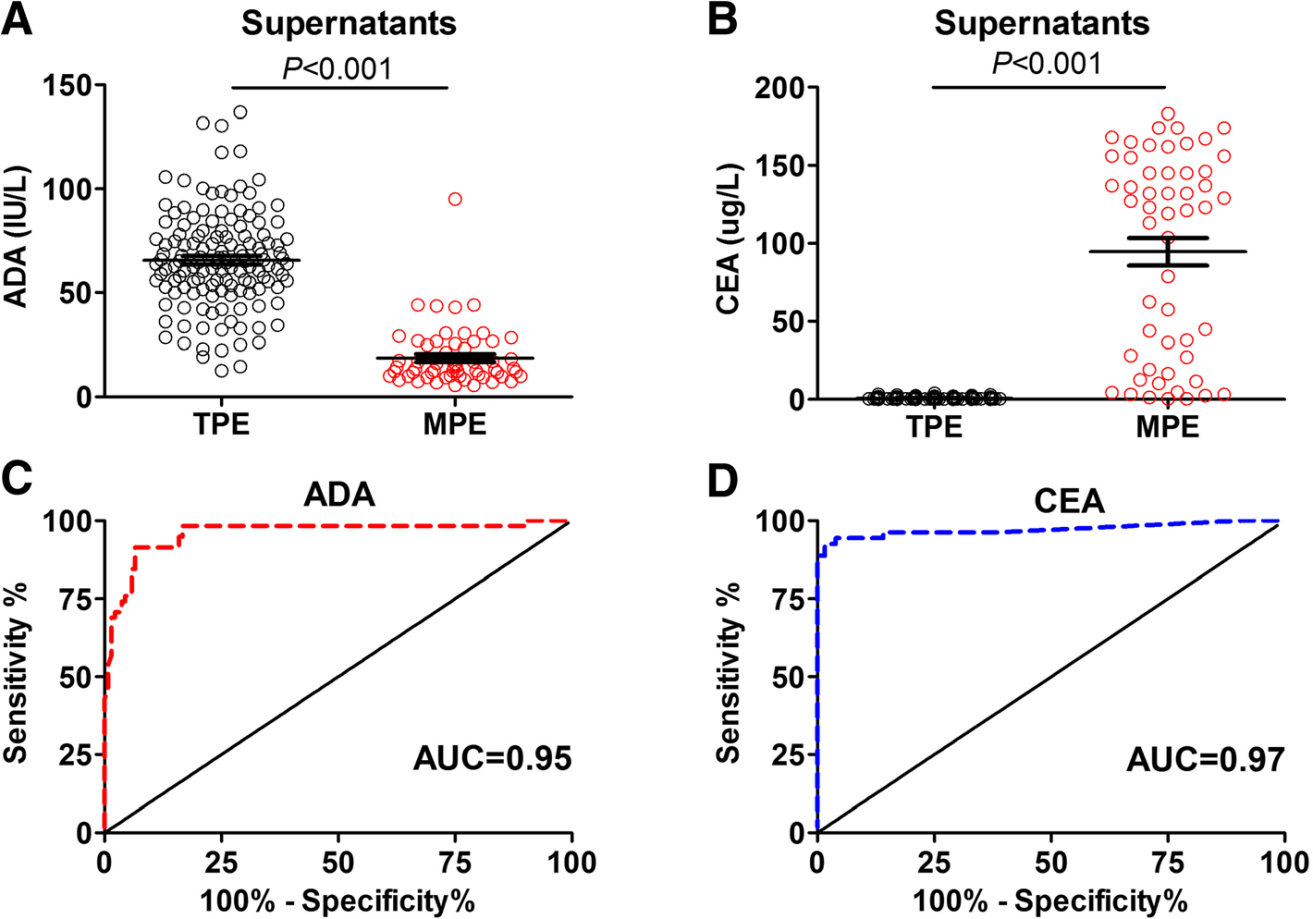
The level of pleural ADA and CEA is useful to discriminate TPE and MPE. **a** After centrifuge of pleural effusion from TPE (*n* = 143) and MPE (*n* = 58) patients, the supernatants was isolated to determine ADA activity using peroxidase method. **b** The supernatants of pleural effusion was used to detect the level of CEA by direct chemilumiscence immunoassay. The unpaired t test was used for the analysis. **c**, **d** ROC analysis was conducted to evaluate the power of ADA (**c**) and CEA (**d**) in discriminating TPE and MPE

Pleural ADA and CEA have been extensively used to discriminate TPE and MPE. Consistently, our results also showed that only the AUC of pleural ADA and CEA were greater than IGRA (Fig. 3). Through the comparison between the level of ADA and CEA in pleural effusion in TPE and MPE categories, pleural ADA median level was significantly higher (*P* < 0.001) in TPE compared to MPE (Fig. 3a). Contrarily, pleural CEA median level was shown to be significantly enriched (*P* < 0.001) in MPE instead (Fig. 3b). The sensitivity and specificity of ADA for discerning TPE from MPE were 93.2 and 89.3%, for CEA, they were 94.3 and 95.1% respectively. The AUCs of the pleural ADA and CEA were 0.95 and 0.97 respectively (Fig. 3c, d).

### Diagnostic evaluations of pleural IGRA, ADA and CEA and their integrations

To further assess the diagnosis efficiency of pleural IGRA combined with ADA and CEA, the corresponding sensitivity, specificity, positive predictive value (PPV), and negative predictive value (NPV) were analyzed (Table 3). The sensitivity and specificity of “pleural IGRA or ADA” for identification of TPE were 96.4 and 29.1%, respectively. The “pleural IGRA or CEA” for MPE diagnosis was determined to have sensitivity and specificity of 99.3 and 31.2%, respectively. Its PPV and NPV values were slightly higher than that of ADA.

**Table 3:** Diagnostic utility of pleural IGRA, ADA, CEA and their integrations for the discriminating diagnosis of TPE and MPE

Assays	TPE		MPE		Sensitivity (95%CI)	Specificity (95%CI)	PPV (95%CI)	NPV (95%CI)
	n	Positivity, n(%)	n	Positivity, n(%)				
Pleural IGRA or ADA	138	133 (96.38)	58	41 (70.69)	0.96 (0.92–0.99)	0.29 (0.18–0.43)	0.76 (0.69–0.83)	0.77 (0.55–0.92)
Pleural IGRA and ADA	138	113 (81.88)	58	1 (1.72)	0.82 (0.74–0.88)	0.98 (0.91–1.00)	0.99 (0.95–1.00)	0.70 (0.58–0.79)
Pleural IGRA or CEA	128	127 (99.21)	53	37 (69.81)	0.99 (0.96–1.00)	0.31 (0.18–0.44)	0.77 (0.70–0.84)	0.94 (0.71–1.00)
Pleural IGRA and CEA	128	111 (86.72)	53	0 (0)	0.87 (0.80–0.92)	1.00 (0.93–1.00)	1.00 (0.97–1.00)	0.76 (0.64–0.85)

Abbreviations: *IGRA* IFN-γ release assay, *ADA* adenosine deaminase, *CEA* arcinoembryonic antigen, *PPV* positive predictive value, *NPV* negative predictive value. The optimal cutoff values of the pleural ADA, CEA, IGRA stimulated with ESAT6 and peptide were 35.0 IU/L, 2.02 μg/L, 87 SFCs and 62.5 SFCs respectively

Nevertheless, when pleural IGRA was combined with either ADA or CEA, it yielded better specificity (98.5 and 100%) and PPV (99.3 and 100%) for the identification of TPE and MPE, respectively. Furthermore, a slightly lower sensitivity for integration with either pleural ADA or CEA was shown, with 82.7 and 87.1% respectively, for TPE and MPE diagnosis. Furthermore, NPV value was equivalent in the integration of pleural IGRA with ADA in comparison to pleural IGRA or ADA alone. Whilst, for the diagnosis of MPE, integration of pleural IGRA and CEA yielded slightly lower value (76.5%) than the combination of “pleural IGRA or CEA”.

### The relationship between pleural adhesion and biomarkers and IGRA in TPE

**Fig. 4:**
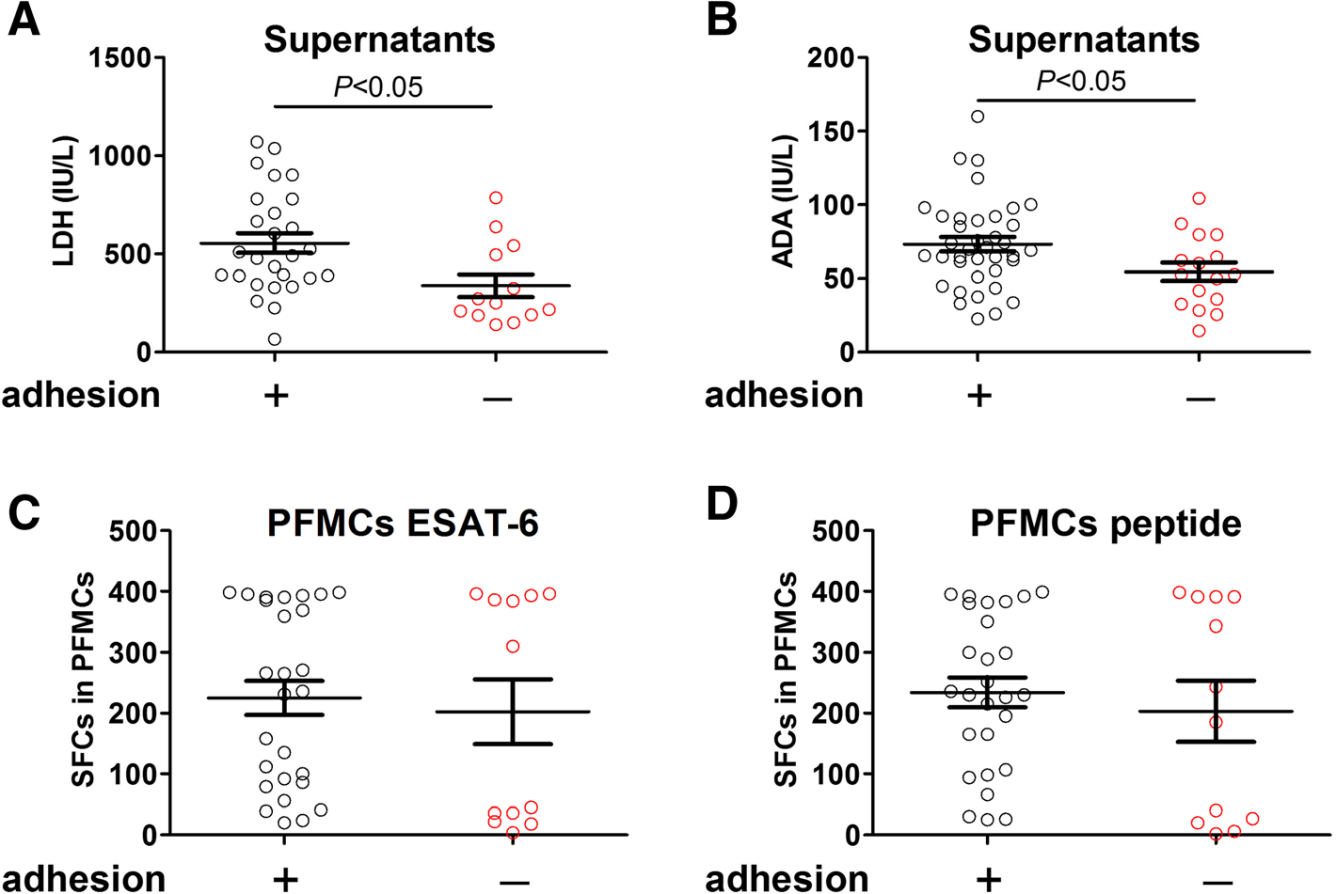
The pleural IGRA doesn’t be affected by adhesion in TPE patients. **a**, **b** The TPE patients were divided into two groups based on pleural biopsy, the levels of LDH (**a**) and ADA (**b**) in the supernatants of pleural effusion were determined, and the differences were compared in TPE patients with and without pleural adhesion. **c**, **d** The PFMCs were stimulated with ESAT-6 protein (**c**) and peptide (**d**) and IFN-γ responses were determined after 24hs incubation, the differences were compared in TPE patients with and without pleural adhesion. The unpaired t test was used for the analysis

To assess the effect of pleural adhesion on the diagnostic performance, the levels of biomarkers (pleural ADA and LDH) as well as pleural IGRA were compared in TPE patients with and without pleural adhesion. As aforementioned, LDH was not statistically significant when compared between TPE and MPE earlier (Table 2). However, the median level of this biomarker (Fig. 4a) was significantly higher (*P* < 0.05) in TPE with pleural adhesion compared to that without pleural adhesion. The same observant was also shown in the median level of ADA (Fig. 4b) which exhibited significant increase in TPE with pleural adhesion. In contrast, both ESAT-6 (Fig. 4c) and peptide (Fig. 4d) induced IFN-γ response did not give any significant difference among TPE patients with and without adhesion, indicating pleural IGRA is a stable approach for differential diagnosis without interference by pleural adhesion.

## Discussion

On a global scale, TB and secondary malignancy remains among the most frequent causes of pleural effusions. Although in both cases, the pleural fluids are primarily composed of lymphocytes, the prognosis and therapeutic management vary considerably [15]. Therefore, discrimination of TPE and MPE is crucial in the medical practice of managing pleural effusion [16]. The TPE diagnosis is normally determined by the manifestation of Mtb in pleural fluid, sputum, or pleural biopsy. Alternately, the effusion as a result of lymphocyte driven immunological response, together with a high ADA level often considered as the inexpensive and easy diagnosis for TPE [17]. However, false-negative and false-positive results remain problematic [18]. Other novel assays might lead to greater diagnostic accuracy, and researchers are working on exploring the specific markers [19].

It is observable that TPE patients have demonstrated greater Mtb antigen-specific IFN-γ responses than MPE patients. For patients with TPE, Mtb antigen-specific IFN-γ responses to ESAT-6 protein and to peptide pool are enriched in pleural effusion in comparison to peripheral blood. A recent study has also reported comparable findings, where higher Mtb antigen-specific IFN-γ reactions were observed in TPE as compared to Non-TB patients, involving MPE, pneumonia and liver cirrhosis [14]. However, other biomarkers, except for ADA, didn’t be evaluated for the discriminating diagnosis of TPE and MPE in that study, and the diagnosis efficiency of combinations of pleural IGRA with other biomarkers also didn’t be assessed. Here, we also found significantly higher levels of ESR, CRP and WCC in TPE patients than in MPE patients, which may assist in differentiating TPE and MPE patients. In contrast, no statistical significance was found for LDH, monocyte %, TP and GLU between TPE and MPE categories. Our study results are comparable to others, except that Duysinx et al. has reported a higher LDH in their study [20].

Among the biomarkers tested for the discriminating analysis of TPE and MPE using the ROC curve, only pleural ADA (0.95) and pleural CEA (0.97) presented greater AUC in comparison to IGRA. The ADA level was higher, while CEA level was lower in TPE than MPE. As such, pleural IGRA, CEA and pleural ADA would be the preferred options to be used to distinguish TPE and MPE patients. However, in the clinical practices, to enhance discriminating diagnostic accuracy and avoid any misdiagnosis, many tests must be taken into account rather than the use of any single method [20]. The integrations of several biomarkers are required to enhance the specificity of the tests. Although IGRA, ADA and CEA were suggested to be highly sensitive when utilized individually for discriminating identification of TPE and MPE, the integrations of IGRA with these 2 biomarkers further improved their specificity. Integration of pleural IGRA with ADA yielded 98.5% specificity, compromising 13.68% sensitivity. In addition, when integrated with CEA, IGRA produced the optimal specificity of 99.36%, with the reduction of only 12.16% sensitivity.

The results suggested that enriched Mtb-specific IFN-γ responses in pleural effusion can also be used as discriminating diagnosis for TPE and MPE. The diagnostic method with higher sensitivity and specificity could lead to enhanced treatment for the patients. Our study demonstrates that the pleural ADA and CEA represent a more effective diagnostic procedure compared to the existing biomarker, such as CRP, LDH, etc. In addition, the integration of IGRA and ADA, CEA provided a promising approach in producing differential diagnoses between TPE and MPE.

## Conclusions

In conclusion, novel pleural effusion biomarkers will contribute to discrimination of TPE and MPE, current evidence suggests Mtb infection elicits elevated IFN-γ responses in the local site of infection, and pleual IGRA can be useful adjuvants for TPE diagnosis, espically combined with ADA and CEA detection. Our data provide a rapid and non-invasive approach in the discernment between TPE and MPE, which is parallel with conventional tests including microbiologic examination and pleural biopsy.

## Abbreviations

ADA: Adenosine deaminase
AFB: Acid-fast bacilli
AUC: Area under curve
CEA: Carcinoembryonic antigen
CFP-10: 10 kDa culture filtrate antigen
CRP: C-reactive protein
ELISPOT: Enzyme-linked Immunospot
ESAT-6: Early secretory antigenetic target of 6 kDa
ESR: Erythrocyte sedimentation rate
GLU: Glucose
IGRA: IFN-γ release assay
LDH: Lactic dehydrogenase
MPE: Malignant pleural effusion
Mtb: *M. tuberculosis*
NPV: Negative predictive value
PBMCs: Peripheral blood mononuclear cells
PFMCs: Pleural fluid mononuclear cells
PHA: Phytohemagglutinin
PPV: Positive predictive value
ROC: Receiver operating characteristic
SFCs: Spot forming cells
TB: Tuberculosis
TP: Total protein
TPE: Tuberculosis pleural effusion
WCC: White cell count
YI: Youden’s index
